# Effect of L-carnitine supplementation on growth performance, nutrient utilization, and nitrogen balance of broilers fed with animal fat

**DOI:** 10.14202/vetworld.2015.482-486

**Published:** 2015-04-12

**Authors:** P. Murali, S. K. George, K. Ally, M. T. Dipu

**Affiliations:** Department of Animal Nutrition, College of Veterinary and Animal Sciences, Mannuthy, Thrissur - 680 651, Kerala, India

**Keywords:** animal fat, broilers, performance and nutrient utilization, l-carnitine

## Abstract

**Aim:**

This experiment was conducted to evaluate the effect of L-carnitine supplementation on growth performance, nutrient utilization and nitrogen balance in broilers fed with animal fat.

**Materials and Methods:**

80 day-old Cobb commercial broiler chicks were randomly assigned into two dietary treatment groups with four replicates of ten chicks each. The diets were isonitrogenous and isocaloric. The birds in both the control (T_1_) and treatment group (T_2_) were fed with a diet having 5% animal fat, while the treatment group (T_2_) was supplemented with 900 mg of L-carnitine. The birds were fed with standard broiler starter ration up to 4 weeks of age and finisher ration up to 6 weeks of age.

**Results:**

The average body weight (g), cumulative feed intake (g) and cumulative feed conversion ratio belonging to groups T_1_ and T_2_ at 6^th^ week of age were 2091.25 and 2151.11, 3976.49 and 4171.68, 1.97 and 1.96 respectively. The percentage availability of the nutrients of two experimental rations T_1_ and T_2_ was 68.23 and 68.00 for dry matter, 58.72 and 55.98 for crude protein, 73.85 and 71.35 for ether extract, 34.19 and 33.86 for crude fiber, 79.18 and 79.59 for nitrogen free extract, 70.24 and 70.03 for energy efficiency and nitrogen balance (g/day) were 2.35 and 2.39, respectively.

**Conclusion:**

This study suggests that the supplementation of 900 mg L-carnitine in diet with added animal fat had no effect on growth performance, nutrient utilization, and nitrogen balance of broilers.

## Introduction

Poultry is one of the fastest-growing segments of the agricultural sector in India, with an average growth rate of 8-10% per annum. Over the last five decades, meat-type chickens have been intensively selected for body weight gain. This selection strategy has resulted in a greater growth rate with improved feed conversion efficiency. However, excessive fat deposition is one of the undesirable consequences of selection for increased growth of modern broiler chickens by the use supplemental fat and oils. Feeding diets with supplemental fat to poultry can have distinct economic advantages by providing increased energy levels at a lesser cost. This is becoming a common practice in poultry production [1]. While, accumulation of fat in carcasses of broilers, particularly in visceral and abdominal areas which represents a waste product to consumers, who are increasingly concerned about the nutritional and health aspects of their food. For this reason, nutritionists continuously try to mediate this problem by means of dietary manipulations, in order to achieve the desired characteristics of growth and carcass composition.

Carnitine has gained interest in the recent years as a potential feed additive for improving chicken meat production and also as a substance with possibly ergogenic properties for increasing physical performance. Carnitine is a quaternary amine (β-hydroxy γ-trimethylaminobutyrate), which is easily soluble in water and found in two stereoisomeric forms, D- and L-carnitine [2]. L-carnitine is synthesized from methionine and lysine almost exclusively in the liver of animals and it plays a key role in energy metabolism of cells, mainly by transferring long-chain acyl groups from cytoplasm to mitochondrial matrix for oxidation by the fatty acid oxidation complex. For years, L-carnitine requirement was not considered due to endogenous biosynthesis. However, studies show that it becomes an essential nutrient under certain circumstances, such as limited carnitine biosynthesis in young animals, diets low in carnitine content, conditions of stress, higher performance, and diets rich in fat [3].

L-carnitine is a vital micronutrient required for lipid metabolism and energy production for poultry. Furthermore, dietary L-carnitine supplementation may have a beneficial effect on broiler nutrition status, mainly due to its sparing effect on its precursor’s lysine and methionine. Providing sufficient amount of L-carnitine to broilers resulted in more efficient utilization of dietary energy and protein [4]. L-carnitine acts by reducing the availability of lipids for peroxidation through transportation of fatty acids into the mitochondria for β-oxidation to produce ATP energy [5]. In addition, L-carnitine through its antioxidant properties increases the levels and activity of antioxidant enzymes such as glutathione peroxidase and superoxide dismutase in the plasma of poultry. Rabie *et al*. [6] found that diet supplemented with L-carnitine from 20 to 60 mg/kg improved the growth performance of broiler chickens. Similarly, supplementation of L-carnitine at 300 mg/kg in the diet with animal fat significantly improved the body weight gain, feed intake and feed conversion ratio in broiler chicken [3]. Gropp *et al*. [7] stated that supplementation of L-carnitine in feed could improve fatty acid and energy utilization and therefore, live weight gain and feed conversion efficiency may be improved in poultry.

Therefore, this study was designed to investigate potential effects of L-carnitine supplementation on the growth performance, nutrients utilization and nitrogen balance in broilers fed with animal fat.

## Materials and Methods

### Ethical approval

The experiment was carried out according to the National regulations on animal welfare and Institutional Animal Ethical Committee.

### Study design

The experiment was conducted using 80 day old unsexed commercial broiler chicks (Vencobb) and the birds were allotted to two treatment groups (T_1_ and T_2_) with four replications of ten chicks each in a separate pen. The dietary treatments were control group (T_1_) fed with standard broiler chicken ration as per BIS [8] specifications with 5% animal fat and the second treatment group (T_2_) were supplemented with L-carnitine at 900 mg/kg diet containing 5% animal fat. The ingredient and chemical composition of the two different broiler starter and finisher rations are presented in Tables-1 and 2. The birds were fed with standard broiler starter ration up to 4 weeks of age and finisher ration up to 6 weeks of age. All birds were maintained under identical management conditions. Feed and clean drinking water were provided *ad libitum* in all the pens throughout the experimental period.

**Table-1:** Ingredient composition of broiler starter and finisher ration, %.

Ingredients	Broiler starter rations, %		Broiler finisher rations, %	
	
	T1	T2	T1	T2
Maize	40	40	48.5	48.5
Soybean meal	41.4	41.4	32.89	32.89
Wheat bran	9	9	9	9
Animal fat	5	5	5	5
Dicalcium phosphate	2	2	2.1	2.1
Calcite	1.79	1.79	1.8	1.8
DL-methionine	0.14	0.14	0.04	0.04
Choline chloride	0.1	0.1	0.1	0.1
Trace mineral mixture	0.01	0.01	0.01	0.01
B complex vitamins	0.01	0.01	0.01	0.01
Vitamin-AB_2_D_3_K	0.1	0.1	0.1	0.1
Toxin binder	0.1	0.1	0.1	0.1
Coccidiostat	0.05	0.05	0.05	0.05
Liver supplement	0.05	0.05	0.05	0.05
Salt	0.25	0.25	0.25	0.25
Total	100.00	100.00	100.00	100.00
To 100 kg of the above mixture, following are added				
L-Carnitine (mg/kg)	-	900	-	900

Trace mineral mixture containing manganese sulfate-60 g, zinc sulfate-50 g, ferrous sulfate-40 g, iodide-2 g, copper-5 g, cobalt-2 g and selenium-0.3 g. B complex vitamins containing vitamin B_1_-8 mg, vitamin B_6_-16 mg, vitamin B_12_-80 μg, vitamin E_50_-80 mg, niacin-120 mg, folic acid-8 mg, pantothenate-80 mg and calcium-86 mg. Vitamin-AB_2_D_3_K vitamin A-82,500 IU, vitamin B_2_-52 mg, vitamin D_3_-12,000 IU, vitamin K-10 mg, calcium-166 mg and phosphate-395 mg. Carniking^®^ (Lonza Group Ltd., Muenchensteinerstrasse, Switzerland) containing lab grade L-carnitine

**Table-2:** Chemical composition of broiler starter and finisher rations*.

Parameters	Broiler starter ration	Broiler finisher ration
Dry matter, %	86.83	87.10
Crude protein, %	23.25	20.14
Ether extract, %	5.48	5.73
Crude fiber, %	4.38	4.16
Nitrogen free extract, %	57.47	62.09
Total ash, %	9.42	7.88
Acid insoluble ash, %	1.90	1.25
Calculated values		
Metabolisable energy, kcal/kg	2805.22	2900.19
Lysine, %	1.27	1.07
Methionine, %	0.34	0.31

* On dry matter basis

The body weight and feed intake of birds were recorded at weekly intervals throughout the experiment to study the pattern of growth under two dietary treatments. Feed conversion ratio (kg of feed consumed/kg body weight gain) was calculated based on the data on body weight gain and feed intake.

### Metabolism trial

A metabolism trial of 3-day duration was conducted after the feeding trial using one bird from each replicate selected randomly. Birds were housed in individual metabolism cages with facilities for feeding, watering, and excreta collection to determine the availability of nutrients and percentage retention of minerals of the experimental rations. Water was provided *ad libitum*. Before the commencement of the actual collection period, birds were subjected to a preliminary adaptation period of 2 days during which they were fed the same quantity of the feed. Excreta were collected for 3 consecutive days over 24 h period using total collection method as described by Summers *et al*. [9]. Excreta collected daily from each bird were weighed and representative samples were taken after thorough mixing. The total amount of feed consumed was also recorded.

The chemical composition of experimental rations and faecal samples was determined as per the AOAC [10]. From the data obtained on total intake and outgo of nutrients during the metabolism trial, availability of nutrients and nitrogen retention were calculated.

### Statistical analysis

Data collected on various parameters were statistically analyzed by completely randomized design as described by Snedecor and Cochran [11]. Means were compared by independent samples *t*-test.

## Results and Discussion

The average body weight gain (g), average feed consumption (g) and feed conversion ratio are presented in Table-3. In both the starter period (0-4 weeks), finisher period (5-6 weeks), and throughout the experimental period (0-6 weeks), L-carnitine supplementation did not affect body weight, feed consumption and feed conversion efficiency in broilers compared to the control group. Similarly, Kheiri *et al*. [12] noticed that feeding diets supplemented with L-carnitine did not affect the performance of broiler chickens. Likewise, Daskiran *et al*. [13] observed that L-carnitine at 150 ppm did not affect feed intake and feed conversion ratio of layers over the 28 days of the experimental period. Supplementation of L-carnitine at 125, 250 and 500 ppm through feed in quails did not affect the feed intake [14]. In this study, dietary L-carnitine did not cause any significant changes in feed intake. This may be because poultry are able to compensate their feed intake according to the energy density of the diet and in this research, the rations had similar energy. Thus, similar feed intake should be maintained over a range of dietary L-carnitine levels.

**Table-3:** Average body weight gain (g), average feed consumption (g) and feed conversion ratio of birds maintained on two dietary treatments.

Week	Average body weight gain^†^, g		p value	Average feed consumption^†^, g		p value	Feed conversion ratio^†^		p value
		
	T1	T2		T1	T2		T1	T2	
Starter (0-4 weeks)	1112.25±26.45	1134.48±17.64	0.49	1854.99±64.34	1925.71±93.05	0.125	1.67±0.08	1.70±0.11	0.30
Finisher (5-6 weeks)	919.22±43.71	989.31±32.68	0.62	2121.50±40.64	2245.96±136.52	0.109	2.33±0.14	2.27±0.08	0.46
0-6 weeks	2031.47±64.62	2123.79±38.45	0.50	3976.49±103.61	4171.68±217.94	0.06	1.97±0.10	1.96±0.10	0.79

† Mean of four values with SE. SE: Standard error

However, Oladele *et al*. [15] stated that supplementation of L-carnitine at 60 ppm either in water or in feed increased the body weight, feed intake and better FCR in broilers. Similarly, Growth performances of broilers were improved by supplementation of L-carnitine at 300 ppm in diet with 5% animal fat [3].

**Figure-1:**
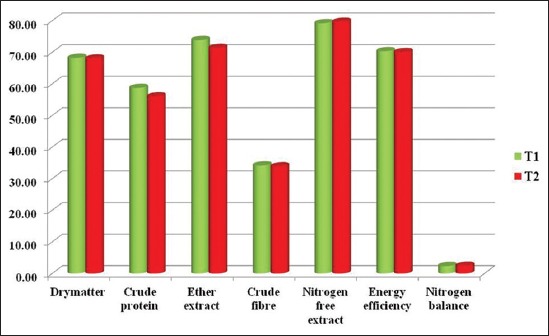
Nutrient availability (%) and nitrogen balance (g/day) of two experimental ration.

The percentage of availability of nutrients and nitrogen balance (g/day) of two experimental rations are depicted in Table-4 and graphically shown in Figure-1. The data on availability of nutrients were statistically similar (p>0.05) between the treatment and control groups. This is in agreement with the findings of Rodehutscord *et al*. [16], who could not find any effect on the availability of crude protein by supplementing L-carnitine at 80 ppm in broiler diet. Han and Thacker [17] also observed that the availability of dry matter, organic matter, crude protein, and crude fiber were unaffected by 50 ppm of L-carnitine in finishing pigs. Ardekani *et al*. [18] stated that supplementation of L-carnitine did not affect the nitrogen excretion in broilers.

**Table-4:** Nutrient availability (%) and nitrogen balance (g/day) of two experimental ration.

Treatments	Dry matter (%)	Crude protein (%)	Ether extract (%)	Crude fibre (%)	Nitrogen free extract (%)	Energy efficiency (%)	Nitrogen balance (g/day)
T_1_	68.23±3.21	58.72±2.92	73.85±3.29	34.19±3.06	79.18±3.08	70.24±3.04	2.35±0.09
T_2_	68.00±1.47	55.98±2.78	71.35±2.31	33.86±1.08	79.59±0.90	70.03±1.31	2.39±0.21
p value	0.48	0.31	0.68	0.81	0.60	0.98	0.25

^†^Mean of four values with SE. SE: Standard error

In contrary to above studies, Beer and Coon [19] stated that L-carnitine supplementation at 50 mg/kg of diet, significantly improved the energy utilization in broiler breeder hens. Whereas availability of dry matter, crude protein and crude fat were increased, when Cho *et al*. [20] supplemented L-carnitine (1000 mg/kg) and lysine (1.60%) in the grower pigs diet. Similarly, Cho *et al*. [21] also observed improvement in availability of dry matter, crude protein and ash with 0.5% L-carnitine in diet of weaned pigs.

Supplementation with L-carnitine could improve the use of dietary nitrogen, either directly through sparing its precursors for protein biosynthesis and other cellular functions or indirectly by optimizing the balance between essential and non-essential amino acids within the cell. Since L-carnitine facilitate the transport of long-chain fatty acids and the removal of short chain and medium chain fatty acids that accumulate as a result of normal and abnormal metabolism [15].

## Conclusions

Different responses by dietary supplementation of L-carnitine are obtained from various studies mainly due to the variations in the species, sex, age, nutrient composition of the diet, levels of L-carnitine in the diet and other environmental conditions. In the present study, data revealed that supplementation of L-carnitine in diet with animal fat had no effect on growth performance in starter periods (0-4 weeks), finisher periods (5-6 weeks) and overall experimental period (0-6 weeks), nutrient utilization, and nitrogen balance of broilers.

## Authors’ Contributions

SKG and KA were involved in the design of the study. PM carried out the experiment and collection and analysis of the data and prepared the first draft of the manuscript under the guidance of SKG and MTD. KA and SKG revised the manuscript. All authors read and approved the final manuscript.
